# Indirect Measurement of Loading Forces with High-Speed Camera

**DOI:** 10.3390/s21196643

**Published:** 2021-10-06

**Authors:** Krzysztof Mendrok, Ziemowit Dworakowski, Kajetan Dziedziech, Krzysztof Holak

**Affiliations:** Department of Robotics and Mechatronics, AGH University of Science and Technology, al. Mickiewicza 30, 30-059 Krakow, Poland; zdw@agh.edu.pl (Z.D.); dziedzie@agh.edu.pl (K.D.); holak@agh.edu.pl (K.H.)

**Keywords:** load identification, load monitoring, vision measurements, high-speed camera

## Abstract

In the last few decades, there has been a significant increase in interest in developing, constructing, and using structural health monitoring (SHM) systems. The classic monitoring system should, by definition, have, in addition to the diagnostic module, a module responsible for monitoring loads. These loads can be measured with piezoelectric force sensors or indirectly with strain gauges such as resistance strain gauges or FBG sensors. However, this is not always feasible due to how the force is applied or because sensors cannot be mounted. Therefore, methods for identifying excitation forces based on response measurements are often used. This approach is usually cheaper and easier to implement from the measurement side. However, in this approach, it is necessary to use a network of response sensors, whose installation and wiring can cause technological difficulties and modify the results for slender constructions. Moreover, many load identification methods require the use of multiple sensors to identify a single force history. Increasing the number of sensors recording responses improves the numerical conditioning of the method. The proposed article presents the use of contactless measurements carried out with the help of a high-speed camera to identify the forces exiting the object.

## 1. Introduction

**Figure 1:**
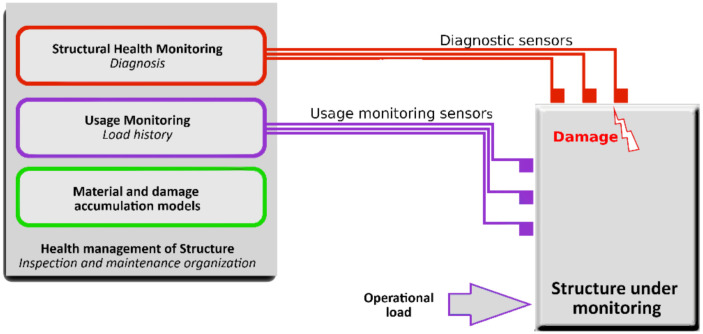
Scheme of an SHM system structure.

Loads affecting a structure during an operation cause wear of its elements. It is crucial from the structure’s durability point of view to monitor and control these loads to assess the level of wear on an ongoing basis or be able to control the object in such a way as to minimize the wear. During the operation of most structures, direct measurement of loading forces is technically difficult or sometimes even impossible, for example, due to the way they are applied or their nature. Therefore, load identification methods have been developed that allow a load to be assessed by measuring the system response. The need to monitor forces acting on exploited objects has been included in the general scheme of the SHM system. According to the book by Balageas et al. [1], the overall diagram of the monitoring system should be in the form shown in Figure 1 and consist of a diagnostic module that detects damage, a load monitoring module that continuously measures the loads, and a predictive module, which, based on information from the former two modules, estimates the remaining lifetime of the object.

These loads can be measured with piezoelectric force sensors or indirectly with strain gauges such as resistance strain gauges or FBG sensors. However, this is not always feasible due to how the force is applied or because sensors cannot be mounted. Therefore, methods for identifying excitation forces based on response measurements are also used. This approach is usually cheaper and easier to implement from a measurement side. However, in this approach, it is also necessary to use a network of response sensors, whose installation and wiring can cause technological difficulties and modify the results for slender constructions. Moreover, many load identification methods require the use of multiple sensors to identify a single force history. Increasing the number of sensors recording responses improves the numerical conditioning of the method. One of the most recent reviews on the topic of excitation forces identification can be found in [2]. Other articles comparing methods for identifying forces are the works by Sanchez and Benarova [3] and Uhl [4].

Vision-based methods are becoming crucial in the non-contact measurement of displacement and velocities of engineering structures [5,6,7,8,9,10,11,12,13,14,15,16,17,18,19,20,21]. However, the use of special high-speed imaging technology is necessary if the dynamic response contains high frequencies, which cameras cannot correctly capture with standard frame rates. High-speed cameras (HSCs), originally used in crash tests in the automotive industry, have found many applications in various branches of science and engineering. Nevertheless, impact studies are one of the most important areas of HSC use. For example, Steinbauer et al. [5] presented an analysis of hailstone impact on an external thermal insulation composite system. Videos of steel and iceball impacts were recorded by a Y4 high-speed camera with a frame rate of 6000 fps.

As indicated above, HSCs were used in research into fracture mechanics, with a recording of crack initiation and propagation. Hosaka et al. [6] applied HSC for resin-dentin interface failure dynamics under tensile loads. An ultra-high-speed Phantom v160 camera was used. Its main parameters are a maximum frame rate of 291,666 fps and a resolution of 256 × 112 pixels. Crack propagation was observed, and failure modes were identified. Liu et al. [7] carried out low-speed impact experiments using a digital 3 × 3 array of high-speed cameras and a 3 × 3 array of LEDs, designed based on Cranz–Schardin’s theory. Each camera in the array was a NET-G1024B CCD camera with a 659 × 494 pixel resolution. The system was used for impact-induced crack initiation and propagation analysis.

Another important application of HSC is fluid flow visualization and analysis, used mainly in the fuel industry and microfluidics. In the paper [8], the authors showed a dual-camera high-speed imaging setup applied to analyze fuel combustion in a shock tube. The setup consisted of two Photron Fastcam SA-X2s, both capable of a 50,000 fps frame rate. It provided visualization of fuel ignition in three dimensions. Ding et al. [9] investigated the dynamic development of nozzle spray patterns of fuel injectors. The ultra-high-speed imaging setup applied in the research consisted of a Shimadzu HPV-2 with a frame rate equal to 1,000,000 fps equipped with a long-distance microscope lens. Znamenskaya et al. [10] discussed the test results of fast-flowing fluids stream ejected from a nozzle at high pressure. The research was aimed at biphasic flow investigation for the water treatment industry. A Photron FASTCAM SA5 high-speed camera with a frame rate of one million fps was used in the experiments. Besides fluid dynamics, HSCs are applied in various investigations. In the field of agriculture, Beczek et al. [11] applied high imaging technology to quantify the soil splash phenomenon, which initiates a water erosion process. A Phantom Micro M310 high-speed camera was used as a recording device. The particle tracking velocimetry (PTV) volumetric method was used to calculate trajectories of soil particles. Ninagawa et al. [12] applied a Phantom camera with a frame rate of 2000 fps and resolution of 512 × 512 pixels in the food industry to investigate intercellular ice crystal formation under different cooling rates. The formation of ice crystals on the epidermal tissue of strawberry geranium leaves was analyzed. In geological research, Song et al. [13] presented observation results of hydrate particles formation in the microflow of water and methane systems. In the experiment, the authors used a Photron FastCam SA-X2 camera with 1024 × 1024 pixel resolution. The frame rate ranged from 250 to 2000 fps.

A lot of recent applications of HSC concerns vibration analysis and structural dynamics. The authors of the paper [14] presented an analysis of foil vibration in a gas foil bearing at high rotational speeds. A Phantom high-speed camera with a maximal frame rate of 12,800 fps was used in the investigation. Tong et al. [15] described a videogrammetry method for three-dimensional vibrations reconstruction of a structure excited on a large-sized shaking table. The tests were carried out to examine the behavior of laminated rubber bearings used to mitigate seismic damage. CamRecord 1000 × 2 (CR1000 × 2) high-speed monochrome CMOS cameras with a resolution of 1280 × 1024 pixels and frame rates of 300 fps were used. HSC data is necessary for vision-based modal analysis. However, modal identification is often difficult due to the noise level at high-frequency components of a vibrational response. Javh et al. [16] addressed this phenomenon by introducing the complex frequency method combined with the least squares frequency domain method to correctly identify high frequencies by incorporating a more precise sensor. An accelerometer is used to identify the eigenvalues, while the camera video data is used to produce the full-field mode shapes. The data were recorded at 200,000 fps with an image resolution of 1024 × 64 pixels.

Another approach to full-field FRF estimation from noise high-speed camera data was presented by Bregar et al. [17]. The identified mode shapes from the least squares frequency domain method and accelerometer data were used to improve the experimental estimation of full-field FRFs using a dynamic substructuring approach. The beam vibration was investigated using a Fastcam SA-Z high-speed with a 200,000 frame rate and 1024 × 48 image resolution. Zhang et al. [18] presented a dynamic vibration analysis using two fast motion extraction algorithms: the modified Taylor approximation refinement algorithm and the localization refinement algorithm to obtain subpixel displacement data. The camera’s frame rate was 1 kH with an image resolution of 300 × 300 pixels. Morimotio et al. [19] presented an active high-speed imaging system consisting of a high-speed camera and a grating projector. The one-pitch phase analysis (OPPA) method was used to determine the phase at every point of a single image for 3D motion and structure reconstruction. The authors presented an application of the developed method: real-time human motion capture and modal analysis of a cantilever beam’s free vibration. For the vibration measurement, video data were captured with a frame rate of 2000 fps.

The following work presents the application of one of the known algorithms to identify forces based on the response signal. A novelty here is the use of a fast camera for measuring the response. Consequently, the force measurement can be carried out completely contactless. It can be implemented from a distance, and the measuring installation does not affect the system’s behavior in any way. Identification of forces by the method of frequency response functions (FRFs) matrix inversion [22] implemented for the displacements measured by a high-speed camera on a laboratory stand is now described.

## 2. Frequency Response Functions Matrix Inversion

Making the assumptions of: linearity, stationarity, and satisfying the reciprocity principle for a considered mechanical system, one can express the response spectrum *x_i_(ω)* in the *i*-th measuring direction excited by the force vector *f(ω)* by the equation:

(1) $$x_{i}\left(\omega \right)=\overset{m}{\underset{j=1}{\sum }}H_{ij}\left(\omega \right)\cdot f_{j}\left(\omega \right)$$

Performing the measurements of responses spectra *x(ω)* in *n* measuring points/directions, one can identify the excitation forces vector *f(ω)* by calculating the pseudoinverse matrix to the matrix *H(ω)* (with dimensions *n* × *m*) [22] according to the formula:

(2) $$\left\{f\right\}\left(\omega \right)=\left[H^{-1}\right]\left(\omega \right)\cdot \left\{x\right\}\left(\omega \right)$$

Overdetermination of the problem (greater number of responses signals than identified forces, *n* ≥ *m*) and application of a singular value decomposition allows for improving the numerical conditioning of the pseudoinverse FRFs matrix calculation.

The FRF matrix is usually obtained from the measurements (e.g., impulse modal test) or from the finite element model [22]. The third possibility to form this matrix of frequency characteristics is their synthesis based on an identified modal model [23]. In the second and third cases, the additional model has to be created or identified to perform FRF synthesis. This makes the method more complex but is sometimes necessary when it is impossible to estimate FRFs from measurements.

The error analysis for the method was investigated by Lee and Park [24]. According to the paper, the biggest inaccuracies occur at the resonance or antiresonance frequencies, depending on the FRF estimation method.

In the described case, FRFs in the form of dynamic flexibility were estimated between the excitation force measured by a piezoelectric force sensor and the displacement of selected points of the object measured by a fast camera. As a reference, the acceleration measurements were used. For that case, the FRFs in the form of inertance were estimated.

## 3. Analysis of the Impact of the Number of Responses on the Identification Accuracy

Based on numerical data, an analysis of the influence of the number of responses included in the identification algorithm on the accuracy of force estimation was carried out. This analysis is crucial for the advisability of using high-speed cameras to identify forces. Measurement with accelerometric sensors allows for collecting about 10–20 responses. Larger numbers are no longer economically justified and may cause hardware problems. In small and slender structures, a greater number of contact sensors also causes a significant structural modification of the measured object.

In order to perform the analysis, a finite element model of a beam similar to the object used in the experimental tests was prepared. The modeled beam was made of aluminum with cross-sectional dimensions of 10 × 30 mm and a total length of 870 mm. The beam ends (80 mm) were clamped with steel blocks and fixed at both ends.

Based on the geometrical model of the test structure, a numerical model was prepared to perform initial analyses of the mode shapes synthesize frequency characteristics.

**Figure 2:**
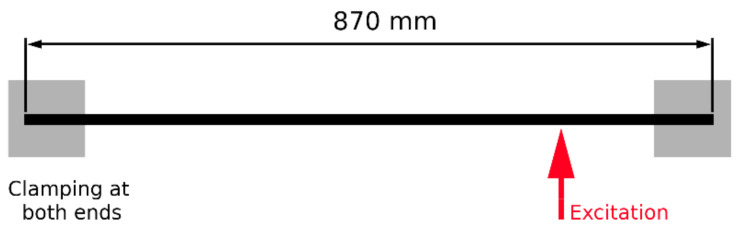
Schematic of the analyzed beam structure.

The geometry of the beam was modeled using an MSC. Patran FE preprocessor and discretized using 8000 3D linear hexahedral elements. There were six elements across the thickness of the beam. The model is shown in Figure 2. The analysis involved the following material parameters: E = 70 GPa, ν = 0.33, and ρ = 2.7 g/mm^3^, where E is Young’s modulus, ν is the Poisson’s ratio, and ρ is the density.

Natural frequencies and mode shapes of the beam were obtained by solving the free undamped vibration problem. Several techniques can be applied for the solution of an eigenproblem. The described study used the Lanczos algorithm, as it is proven to accurately compute a discrete set of eigenvalues and eigenvectors for medium and large-size FE models. It is also important to note that the algorithm used does not miss any roots and offers very good numerical performance.

The MSC.Nastran FE solver was used to perform computations. The normal modes solution (SOL103) was applied to determine the vibration mode shapes of the frame in both damaged and undamaged states.

The obtained modal parameters were used to synthesize FRFs for 2193 beam nodal points in the direction normal to the plane defined by the longer beam dimensions (vertical direction in Figure 2). The following formula was used to synthesize FRF:

(3) $$H_{ij}\left(\omega \right)=\overset{n}{\underset{r=1}{\sum }}\frac{2j\omega_{r}\phi_{ri}\phi_{rj}}{\omega_{r}^{2}-\omega^{2}}$$

where: *n* is the number of modes taken for analysis, *ω_r_* is the *r*-th natural frequency, *φ_ir_* is the *r*-th modal vector element related to *i*-th response location, and *φ_jr_* is the *r*-th modal vector element related to *j*-th excitation location.

**Figure 3:**
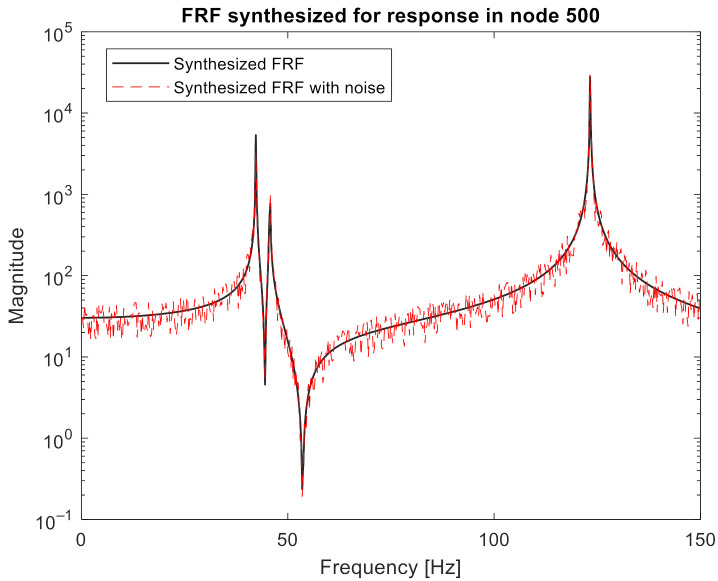
Example of synthesized FRF.

The frequency range 0–150 Hz was considered for the synthesis, which included the first three modes. The frequency resolution was set to 0.25 Hz. The excitation point was selected at node 509. Each of the characteristics was then noised with noise with a normal distribution, mean 0, and a standard deviation of 20% of the mean amplitude of the disturbed characteristic. An example of synthesized characteristics before and after adding the noise is presented in Figure 3.

**Figure 4:**
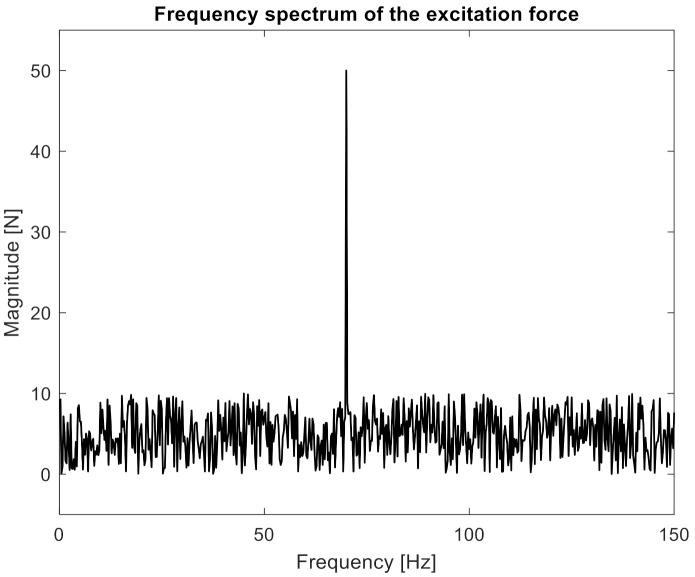
The frequency spectrum of the excitation signal.

Then, the excitation signal was constructed as the sum of the noise with a uniform distribution on the open interval (0, 10 N), sine with a frequency of 70 Hz, and an amplitude of 50 N. In Figure 4, the frequency spectrum of the excitation signal is presented.

By multiplying the excitation signal by FRFs, frequency spectra of 2193 responses were obtained. The responses were also disturbed by noise with parameters identical to the one used for FRFs.

**Figure 5:**
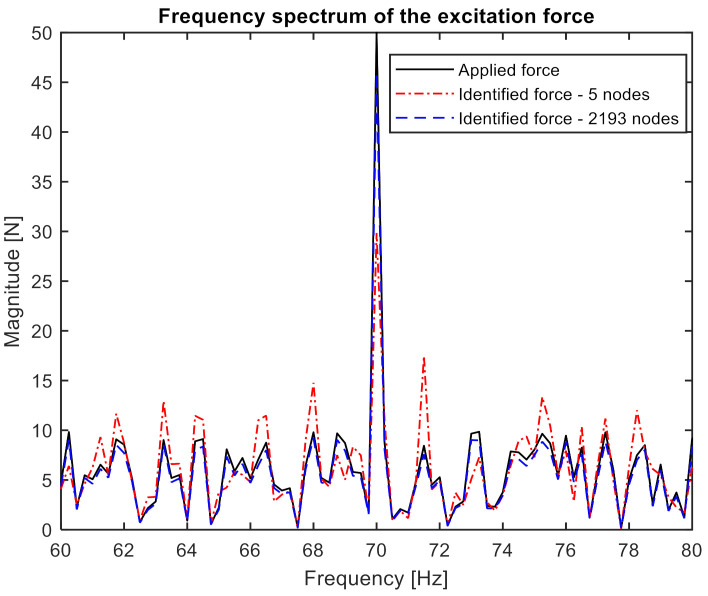
Comparison of the frequency spectrum of the applied and identified force.

The matrix of noised FRFs and the response vector, also noisy, were then used to calculate the exciting force according to Formula (2). For subsequent analyzes, 5, 10, 20, 50, 100, 200, 500, 1000, 1500, and 2193 nodal points were taken, respectively. For each of the cases, nodes were evenly distributed over the length of the beam. Figure 5 compares the applied and identified force for the extreme cases (5 and 2193 responses). In order to show the identification quality more clearly, the presented frequency band was limited to a range of 60–80 Hz.

As a numerical measure of the identification quality, the Pierson correlation coefficient [25] was calculated between the spectrum of the set and identified force and the amplitude at the frequency of 70 Hz, i.e., for the harmonic component of the excitation signal, was used.

In Table 1, the obtained results are presented.

Presented results clearly showed that the number of responses taken to force identification significantly improves the quality of the obtained spectrum. This further justifies the application of vision measurement in this process.

## 4. Experiment Description

**Figure 6:**
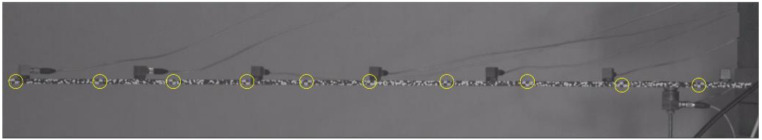
Cantilever beam investigated in the laboratory experiment.

For experimental verification of the possibility of identifying excitation using camera displacement measurements, a laboratory experiment was carried out. The examined structure was a cantilever beam (Figure 2) with dimensions: length 800 mm, width 30 mm, and thickness 5 mm. On the side face of the structure, a set of crash test optical markers was placed. They are marked with yellow circles in Figure 6. Additionally, the side of the beam was covered with optical noise to enable the identification of vibration displacements in a larger number of points. Seven accelerometers were connected to the beam to act as reference measurement devices.

The vision-based measurement system consisted of a high-speed Phantom V9 camera and two halogen lamps. The resolution of the vision sensor was set to 1632 × 800 pixels. Carl Zeiss lens with a focal length of f = 50 mm and an f-number of F/5.6 was used. During the measurement, the frame rate of the camera was set to 1000 Hz. The camera was oriented with respect to the analyzed object so that its optical axis was perpendicular to the object’s side face.

In the experiment, the beam was forced to vibrations at one point by an electrodynamic shaker, The Modal Shop TMS K2007E01. The broad-band noise signal was used to excite all identified natural frequencies. There were two main reasons to select such an excitation signal. First, the authors wanted to reflect the operating conditions. Second, the noise signal has high complexity. The level of the excitation signal amplitude was selected so that the vibrations of the object were measurable by the camera, also for higher frequencies

A high-speed camera recorded the response of the beam. In the post-processing step, displacements of the measurement points in the image plane were computed using a feature tracker available in TEMA Automotive commercial software with an accuracy of 0.05 pixels, taking into account subpixel techniques. The correlation-based tracking algorithm was chosen due to its high robustness to image noise. Displacements were determined at the location of 10 markers and additionally at 90 points evenly spaced along the length of the beam (based on optical noise).

As a reference measuring system, the accelerometers PCB 333B30 were used for the simultaneous measurement of responses. The force signal and vibrations acceleration at the driving point was measured by the impedance head PCB 280D01. Accelerometric measurements and excitation signals were carried out with the help of Test. Lab 10B software. The sampling frequency of the acceleration and force measurements was set at 2048 Hz. Time histories of length 6 s were recorded for both measuring systems.

In the preliminary test, the first four natural frequencies of the beam were identified at 40 Hz, 110 Hz, 209 Hz, and 347 Hz.

## 5. Identification Procedure and Results

**Figure 7:**
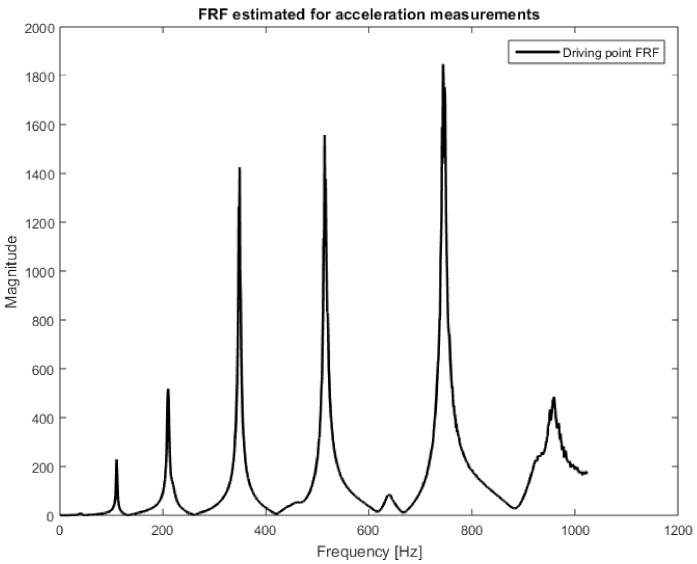
Driving point FRF estimated for the vibrations’ acceleration measurements.

In the first step, the responses measured by accelerometers were used to identify the excitation force. For this purpose, seven FRFs were determined between the excitation force signal and the responses measured by the accelerometers. The H1 estimation method was used with the following parameters: the number of averages, 11; overlapping, 50%; frequency resolution, 1 Hz. At the same time, the spectra of excitation force, and response signals were calculated with the same processing parameters. An example of the magnitude of the estimated FRF is shown in Figure 7.

**Figure 8:**
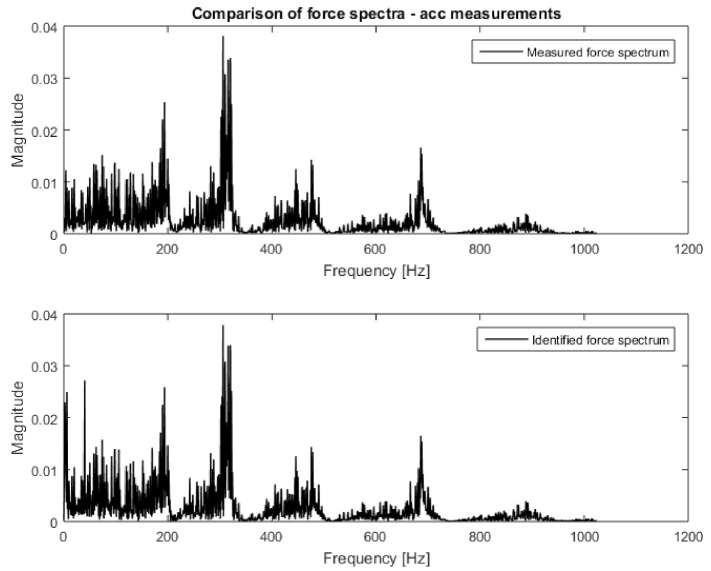
Comparison of measured and identified force spectra for the vibrations’ acceleration measurements (entire frequency range).

**Figure 9:**
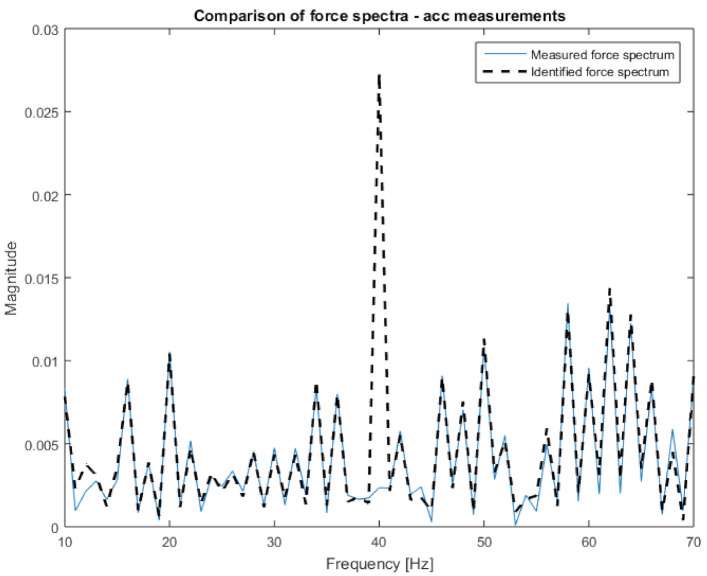
Comparison of measured and identified force spectra for the vibrations’ acceleration measurements (narrowed frequency range).

Then, the data prepared in this way were compiled in the H and x matrices according to Equation (1). Both had a size of 7 × 1. Based on Equation (2), the excitation force spectrum was calculated. The obtained characteristic, together with the measured one, is presented in Figure 8. Both spectra are almost identical; that is why they were placed on separate plots. Additionally, in Figure 9, the narrowed frequency band of the two spectra is shown on one plot to allow for detailed comparison. The zoomed plot shows a good quality of the identified force. The exception is the frequency of 40 Hz, where one of the natural frequencies of the beam is located. There is a significant overestimation of the value of the identified force. This is in line with the analysis of errors from work [22]. It is worth noting that in the other natural frequencies, the identification errors are no longer that large. As a measure of the quality of identification, the value of Pearson’s correlation coefficient between both spectra and the average value of signal spectrum magnitude were assumed. The results for both criteria are presented in Table 2. The research began with analysis using vibration acceleration measurements (these are well described in the literature) to verify the correctness of the adopted solutions in the field of measurements, signal processing, and calculations. After positive verification, attempts were made to apply vision measurements to identify forces.

**Figure 10:**
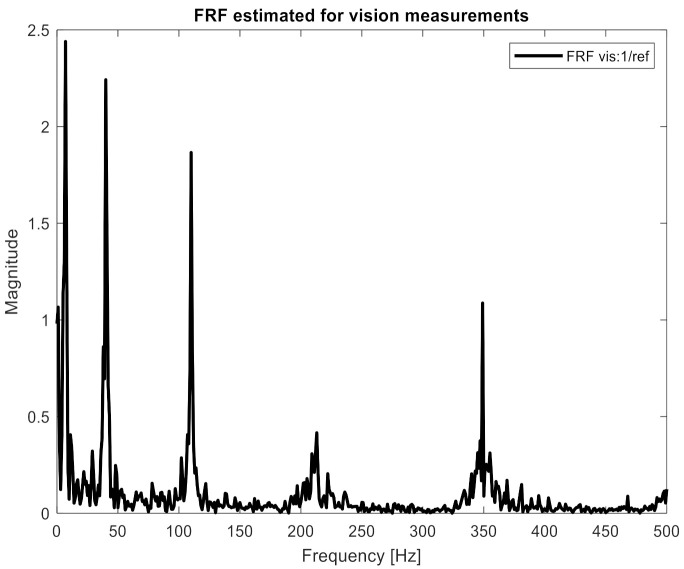
FRF estimated for the vision measurements between excitation force and response measured in point vis:1 (the first marker on the left side of the beam).

The identification procedure was repeated for displacement measurements taken with a high-speed camera. In the first step, it was necessary to unify the sampling frequency of the force (2048 Hz) and response signals (1000 Hz). For this purpose, the force signal was re-sampled to have a sampling frequency of 1000 Hz. Then, it was necessary to synchronize force and response signals in the time domain. The moment the shaker was switched on was used for this task. Next, 90 FRFs were estimated between the excitation force and the responses measured by the vision system. Again, the H1 estimation method was used with the same processing parameters: the number of averages, 11; overlapping, 50%; frequency resolution, 1 Hz. Additionally, the spectra of displacements signals were calculated. The same processing parameters were used. One of the estimated FRFs is presented in Figure 10.

**Figure 11:**
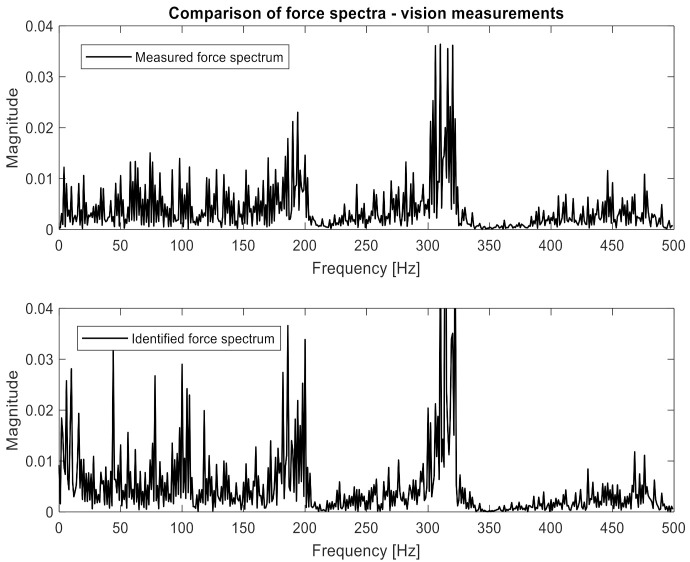
Comparison of measured and identified force spectra for the camera measurements (10 points—entire frequency range).

**Figure 12:**
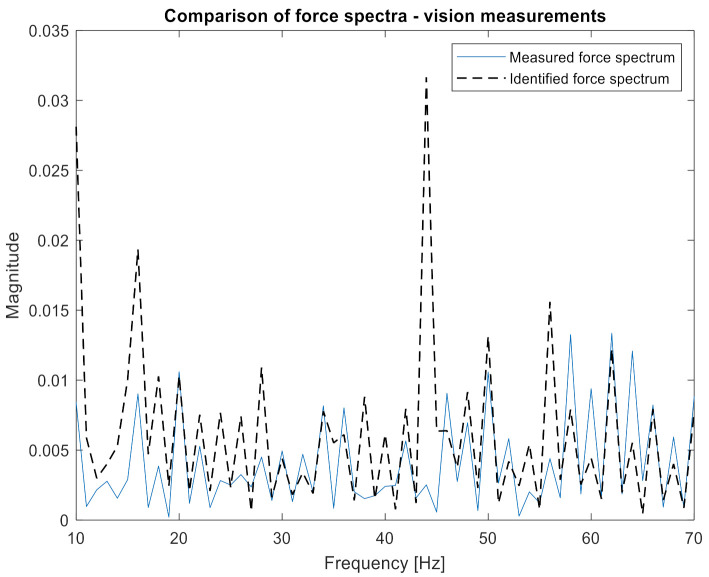
Comparison of measured and identified force spectra for the camera measurements (10 points—narrowed frequency range).

**Figure 13:**
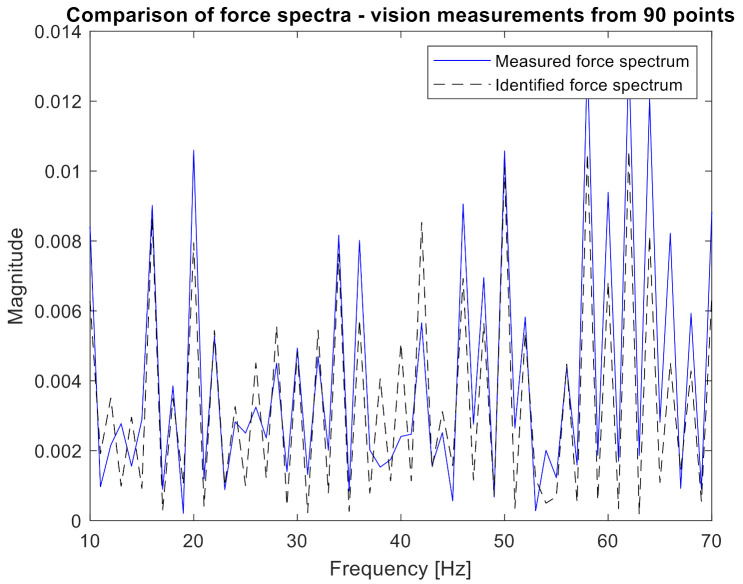
Comparison of measured and identified force spectra for the camera measurements (90 points—narrowed frequency range).

One can clearly observe the worse quality of the obtained characteristic in comparison to measurements performed with accelerometers. Again, the H and x matrices were built with appropriate FRFs and displacement spectra. In order to confirm the relationship between the number of measurement points and the accuracy of identification, 10, 19, 45, and 90 points were applied to the procedure successively. Both the H and x matrices had a size of 10 × 1, 19 × 1, 45 × 1, and 90 × 1, respectively. Inserting them (separately for each frequency) into Equation (2), the excitation force spectrum was identified. The results are presented in Figure 11, Figure 12 and Figure 13. The same visualization method was used to facilitate the interpretation and comparison of results obtained for accelerometer and vision data. Of course, the same identification quality indicators were used. The results for the vision data, together with the previous results, are shown in Table 1.

Figure 11 shows that the nature of the excitation spectrum was correctly reproduced. However, the details of Figure 12 indicate that the quality of identification achieved using vision measurements is worse than with the accelerometric measurements. On the other hand, in Figure 13, where the identification was carried out with the use of 90 measuring points, the quality of the obtained spectrum does not differ from the results obtained for the accelerometers.

Numerical indicators also show a decrease in the accuracy of force identification using vision measurements and a comparable number of measuring points. When the number of points taken for analysis grows, the quality of the identified force spectrum increases significantly. It is worth noting that for 90 measuring points, the same level of spectrum amplitudes was obtained as for the measurement with accelerometers, and the correlation coefficient is only slightly lower. This reduction is due to lower quality in the high frequencies. As a positive of the performed analysis, it can be considered that the value of force was overestimated. It is a safer alternative than underestimating the load as it leads to more frequent than necessary maintenance procedures with a low risk of damage occurrence.

## 6. Summary and Conclusions

The paper shows that it is possible to identify the force acting on a structure based on the response measurement performed with a fast camera. The quality of the obtained results is slightly lower than previously described in the literature identifications obtained based on accelerometric measurements. In the authors’ opinion, the improvement in the quality of force identification can be achieved by:

– extension of response registration time (only 11 averages in the time domain with 50% overlap was used);
– synchronization of both measuring systems—force and responses measurements for FRFs matrix determination (in the presented work, synchronization was performed manually at the post-processing stage);
– unification of recording parameters such as sampling frequency.

However, it is worth remembering that the measurement carried out with the use of a high-speed camera is non-contact; thus, it does not modify the structure and causes no issues with the cabling.

## Figures and Tables

**Table 1:** Quantitative assessment of the identification of forces.

	No. of Nodes	Pearson’s Correlation Coefficient	70 Hz Peak Amplitude [N]	Relative Error [%]
Applied force		1.0000	50,0000	0
Identified force	5	0.8074	34.4028	31.1943
Identified force	10	0.8733	37.6962	24.6076
Identified force	20	0.9223	37.8176	24.3647
Identified force	50	0.9714	41.9471	16.1057
Identified force	100	0.9846	43.9666	12.0668
Identified force	200	0.9932	45.6558	8.6885
Identified force	500	0.9973	44.0192	11.9616
Identified force	1000	0.9987	46.1541	7.6918
Identified force	1500	0.9992	45.9200	8.1600
Identified force	2193	0.9994	46.2998	7.4005

**Table 2:** Quantitative assessment of the identification of forces.

	Pearson’s Correlation Coefficient	The Average Value of Spectrum Magnitude	Relative Error [%]
Measured force		0.0039	
Identified force—acc—7 points	0.95	0.0041	4.2
Identified force—cam—10 points	0.65	0.0049	25.6
Identified force—cam—19 points	0.77	0.0047	20.5
Identified force—cam—45 points	0.80	0.042	7.7
Identified force—cam—90 points	0.82	0.041	4.2

## Data Availability

Data supporting reported results can be accessed after e-mail contact with the first author.
